# Association of perioperative use of statins, metformin, and aspirin with recurrence after curative liver resection in patients with hepatocellular carcinoma: A propensity score matching analysis

**DOI:** 10.1002/cam4.6569

**Published:** 2023-09-22

**Authors:** Elias Khajeh, Ehsan Aminizadeh, Arash Dooghaie Moghadam, Ali Ramouz, Rosa Klotz, Mohammad Golriz, Uta Merle, Christoph Springfeld, De‐Hua Chang, Thomas Longerich, Markus W. Büchler, Arianeb Mehrabi

**Affiliations:** ^1^ Department of General, Visceral, and Transplantation Surgery Heidelberg University Hospital Heidelberg Germany; ^2^ Liver Cancer Center Heidelberg (LCCH) Heidelberg University Hospital Heidelberg Germany; ^3^ Department of Internal Medicine IV, Gastroenterology & Hepatology Heidelberg University Hospital Heidelberg Germany; ^4^ Department of Medical Oncology National Center for Tumor Diseases Heidelberg University Hospital Heidelberg Germany; ^5^ Department of Diagnostic and Interventional Radiology Heidelberg University Hospital Heidelberg Germany; ^6^ Institute of Pathology Heidelberg University Hospital Heidelberg Germany

**Keywords:** aspirin, hepatocellular carcinoma, liver resection, metformin, statin, survival

## Abstract

**Background:**

Statins, metformin, and aspirin have been reported to reduce the incidence of hepatocellular carcinoma (HCC). However, the effect of their perioperative use on survival outcomes of HCC patients following curative liver resection still remains unclear.

**Method:**

Three hundred and fifty three patients with a first diagnosis of HCC who underwent curative liver resection were included. Propensity score matching analysis with a users: nonusers ratio of 1:2 were performed for each of the medications (statins, metformin, and aspirin). Overall survival (OS) and recurrence‐free survival (RFS) were evaluated and multivariable Cox proportional hazard analysis was performed.

**Results:**

Sixty two patients received statins, 48 patients used metformin, and 53 patients received aspirin for ≥90 days before surgery. None of the medications improved OS. RFS of statin users was significantly longer than that of nonusers (*p* = 0.021) in the matched cohort. Users of hydrophilic statins, but not lipophilic ones had a significantly longer RFS than nonusers. Multivariable analysis showed that statin use significantly improved RFS (hazard ratio [HR]: 0.41, 95% confidence interval [CI]: 0.17–0.97, *p* = 0.044). No difference was seen in RFS between metformin users and nonusers. Among patients with diabetes, RFS was nonsignificantly longer in metformin users than in non‐metformin users (84.1% vs. 60.85%, *p* = 0.069) in the matched cohort. No difference in postoperative RFS was seen between aspirin users and nonusers.

**Conclusion:**

Preoperative use of statins in patients with HCC can increase RFS after curative liver resection, but metformin and aspirin were not associated with improved survival. Randomized controlled trials are needed to confirm the findings of the present study.

## 1 INTRODUCTION

Hepatocellular carcinoma (HCC) is the sixth most frequent cancer and the third most common cause of cancer‐related mortality in the world.^1^ Liver resection is the most commonly used curative treatment of HCC.^2^ Advances in surgery and early detection of HCC improved the postoperative HCC survival rate, but the recurrence rate remained high.^3^, ^4^ Therefore, recognizing potent strategies to reduce the recurrence of HCC is crucial. Recent studies tried to explore the potential risk factors of recurrence of HCC after curative resection.^4^, ^5^ The chemopreventive role of common medications such as statins, metformin, and aspirin in reducing the incidence of HCC has already been investigated.^6^, ^7^, ^8^ These studies, along with molecular and experimental studies, have suggested that these drugs affect the oncological outcome of patients with HCC after surgery.^6^, ^7^, ^9^

Previous studies have shown that statins, metformin, and aspirin can improve recurrence‐free survival (RFS) in HCC patients after surgery.^7^, ^9^, ^10^ To the best of our knowledge, all studies investigating the impact of statins, metformin, and aspirin on HCC recurrence after surgery have originated from Asia‐Pacific countries. These results may be different from those in Western populations because demographics and primary cause of cirrhosis and HCC are different between these two populations. Furthermore, the prevalence of HCC is much higher in Asia‐Pacific countries than in Western countries; therefore, different rates of underlying diseases that cause HCC may have different effects in the two populations.^7^ There was also a lack of data regarding the type, dosage, and duration of usage of these medications in previous studies, which may have influenced the outcomes. To address these gaps, we evaluated the effect of perioperative statins, metformin, and aspirin on the survival of patients undergoing liver resection with due attention to the type and dosage of these medications.

## 2 MATERIALS AND METHODS

### 2.1 Study design

We analyzed patients with a first diagnosis of HCC who underwent curative liver resection with negative resection margins between 2002 and 2019 at the Department of General, Visceral, and Transplant Surgery of Heidelberg University Hospital. Data of patients who underwent their first curative treatment were obtained from our prospectively maintained database. Patients who had additional malignancies at the time of diagnosis, who underwent non‐curative liver resection, who had positive resection margins, or who were being operated for recurrent HCC were excluded. The consort flow diagram of the study is presented in Figure S1. The independent ethics committee of Heidelberg University approved the study protocol (S‐754/2018) and all procedures were done according to the principles of the Declaration of Helsinki. All patients signed an informed consent form at admission, allowing their data to be used for study purposes in the future.

### 2.2 Definition of exposure to medications

Patients who received a daily dose of statins, metformin, or aspirin for a duration of at least 90 days before surgery until the time of operation were considered users of the medication. Statins were categorized into lipophilic (atorvastatin, simvastatin, lovastatin, fluvastatin, cerivastatin, and pitavastatin) and hydrophilic (rosuvastatin and pravastatin) medication. The intensity of the statins was categorized according to American Heart Association guidelines as low, moderate, or high intensity.^11^

### 2.3 Outcomes and measurements

#### 2.3.1 Preoperative data

Demographic and preoperative characteristics—including age, sex, body mass index (BMI), American Society of Anesthesiologists (ASA) classification, and medical history of hepatitis B (HBV) or C (HCV), cirrhosis, and diabetes mellitus—were obtained from the liver surgery database. We also extracted the Child‐Pugh score for all patients with cirrhosis. Perioperative use of statins, metformin, or aspirin was evaluated for all patients. Preoperative liver‐specific laboratory results were also extracted.

#### 2.3.2 Intraoperative data

Data regarding extent of the resection, blood loss, and operation duration were collected.

*Postoperative outcomes*: Postoperative data included perioperative complications, recurrence, five‐year mortality, and pathological findings. These pathological findings included the number, location and maximum size of resected tumors, the margin status after resection (R), the TNM classification, and the tumor grade (G). RFS was defined as the time from surgery to the time to recurrence or last follow‐up. Overall survival (OS) was defined as the time from surgery to the date of death or last follow‐up. Recurrence/death free survival was defined as the time from surgery to the time to recurrence, death, or last follow‐up.

### 2.4 Statistical analysis

Dichotomous data are presented as percentages and differences between groups were evaluated using the chi‐square test or Fisher's exact test. Continuous variables are expressed as the mean ± standard deviation (SD) and were evaluated using the *t*‐test or Mann–Whitney test. The Kaplan–Meier method was used to plot the RFS and OS curves stratified by medication use and the log‐rank test was used to compare them. Variables with a *p* value <0.1 in the univariate analysis were included in the multivariable analyses of RFS and OS using Cox proportional hazard analysis. Hazard ratios (HRs) with 95% confidence intervals (CIs) were estimated for each variable. To examine the assumption proportional hazards we added the interaction of time with statin, metformin, and aspirin in the univariable cox regression analysis of these medications in each corresponding propensity matched cohort. Patients who died in the perioperative period were excluded from the survival analysis. Propensity score‐matching analysis was used to build a matched group of patients and to minimize differences in confounders and baseline characteristics between the groups of patients using statin, metformin, or aspirin and nonusers. Age, gender, BMI, liver cirrhosis, hyperlipidemia, cardiovascular disease, diabetes, extent of resection, number of tumors, tumor size, pathological extend (T), and pathological grade (G) of the tumor were chosen as potentially confounding factors. Logistic regression analysis was used to estimate the propensity score. Patients were matched based on the logit of the propensity score by selecting the nearest available matching using a caliper width of 0.1 of the SD of the estimated propensity score between study groups. Accordingly, a 1:2 matched analysis was conducted. A propensity score matching analysis with a users: nonusers ratio of 1:1 was performed among patients with diabetes for metformin. All statistical tests were two‐sided and a *p* < 0.05 was considered significant. Statistical analyses were performed using SPSS 24.0 software (SPSS Inc.) and the propensity score matching algorithm was performed using R, version 3.5.1 (The R Foundation for Statistical Computing).

## 3 RESULTS

### 3.1 Patient characteristics

Of 353 patients with newly diagnosed HCC who underwent curative liver resection, 62 (17.6%) received statins (lipophilic statins in 35 patients and hydrophilic statins in 27 patients), 48 (13.6%) received metformin, and 53 (15.0%) received aspirin before surgery. The median follow‐up of the patients was 27 months (interquartile range: 12–52 months) and the three‐year recurrence rate was 16.4% (58 patients). Among the 58 patients with recurrence, 41 patients had multiple intrahepatic lesions, 14 patients had single intrahepatic lesion, and three patients had extrahepatic recurrence. Table 1 shows the comparison of baseline characteristics, intra‐, and postoperative data between patients with and without recurrence after surgery. No significant differences were seen in baseline data including age, gender, BMI, history of diabetes, hyperlipidemia. cardiovascular disease, HBV and HCV infection, grading of cirrhosis, and ASA class between the groups. The rate of cirrhosis was lower in patients with HCC recurrence (34.5% vs. 48.8%, *p* = 0.045). There was also no significant difference in the proportion of patients with high serum alpha‐fetoprotein (AFP) levels and thrombocytopenia between the groups. Intraoperatively, no significant differences were observed in the extent of liver resection, blood loss, blood transfusion, or operative time between the groups. More patients with recurrence had a pathological grading of G3–4 (17/58, 29.3%) than patients without recurrence did (40/295, 14.5%) (*p* = 0.016). The rate of vascular invasion was also higher in patients with recurrence (60.3% vs. 28.2%, *p* < 0.001). Other parameters of tumor morphology and pathology were not significantly different between the two groups. Postoperative outcomes including major complications, posthepatectomy liver failure (PHLF), posthepatectomy bile leakage (PHBL), and posthepatectomy hemorrhage (PHH) did not differ between groups (Table 1).

**TABLE 1:** Baseline characteristics, intraoperative outcomes, and postoperative outcomes of patients with and without HCC recurrence after curative resection.

	No recurrence (*n* = 295)	Recurrence (*n* = 58)	*p*
*Preoperative data*			
Gender (male), *n* (%)	234 (79.3)	43 (74.1)	0.380
Age, years, mean ± SD	64.1 ± 10.7	62.6 ± 12.9	0.360
BMI ≥30 kg/m^2^, *n* (%)	70 (23.8)	14 (24.1)	0.957
Diabetes mellitus, *n* (%)	88 (29.8)	15 (25.9)	0.543
Hyperlipidemia, *n* (%)	131 (44.5)	26 (44.8)	0.982
Cardiovascular disease, *n* (%)	137 (46.4)	28 (48.2)	0.971
HBV infection, *n* (%)	39 (13.2)	5 (8.6)	0.332
HCV infection, *n* (%)	50 (16.9)	5 (8.6)	0.110
Cirrhosis, *n* (%)	144 (48.8)	20 (34.5)	**0.045**
Child‐Pugh classification			0.352
A, *n* (%)	138 (46.7)	20 (34.3)	
B, *n* (%)	6 (2.0)	0 (0)	
ASA score 3–4, *n* (%)	119 (40.3)	30 (51.7)	0.076
AFP ≥200 ng/mL, *n* (%)	156 (52.9)	28 (48.3)	0.521
Platelet <150/nl, *n* (%)	82 (27.8)	18 (31.0)	0.617
*Intraoperative data*			
Major liver resection, *n* (%)	113 (38.3)	25 (43.31)	0.494
Blood loss, ml, mean ± SD	770.6 ± 710.1	743.6 ± 951.1	0.810
Intraoperative blood transfusion, *n* (%)	36 (13.7)	5 (9.1)	0.355
Operation time > 150 min, *N* (%)	129 (43.7)	28 (48.2)	0.765
*Postoperative data*			
Multiple tumors, *n* (%)	52 (17.8)	16 (27.6)	0.086
Bilobar tumors, *n* (%)	44 (14.9)	9 (15.5)	0.907
Maximum tumor size >3 cm, *n* (%)	207 (70.9)	45 (77.6)	0.300
Tumor stage of T3–T4, *n* (%)	47 (17.4)	16 (27.6)	0.074
High tumor grade (G3–4), *n* (%)	40 (14.5)	17 (29.3)	**0.016**
Vascular invasion, *n* (%)	83 (28.2)	35 (60.3)	**<0.001**
Major complications (Clavien‐Dindo≥3), *n* (%)	41 (13.9)	3 (5.2)	0.112
PHLF, *n* (%)	9 (2.5)	1 (1.7)	1
PHBL, *n* (%)	13 (4.4)	2 (3.4)	1
PHH, *n* (%)	9 (3.1)	1 (1.7)	0.578

The bold values are the p values of those which are statistically significant.

Univariable and multivariable analyses of factors associated with RFS and OS are presented in Table S1. In the multivariable analysis, statin use was an independent protective factor against HCC recurrence after liver resection (HR: 0.42; 95% CI: 0.19–0.94; *p* = 0.036), while a tumor size ≥3 cm (HR: 2.25; 95% CI: 1.14–4.48; *p* = 0.020) and vascular invasion (HR: 2.46; 95% CI: 1.42–4.26; *p* = 0.001) were associated with recurrence after resection. Regarding OS, multivariable analysis showed that an Operative time > 150 min (HR: 3.05; 95% CI: 1.21–7.69; *p* = 0.018) and multiple tumors (HR: 3.49; 95% CI: 1.47–8.28; *p* = 0.005) were independent risk factors associated with reduced OS after liver resection.

### 3.2 The effect of statin use on survival outcomes

**FIGURE 1:**
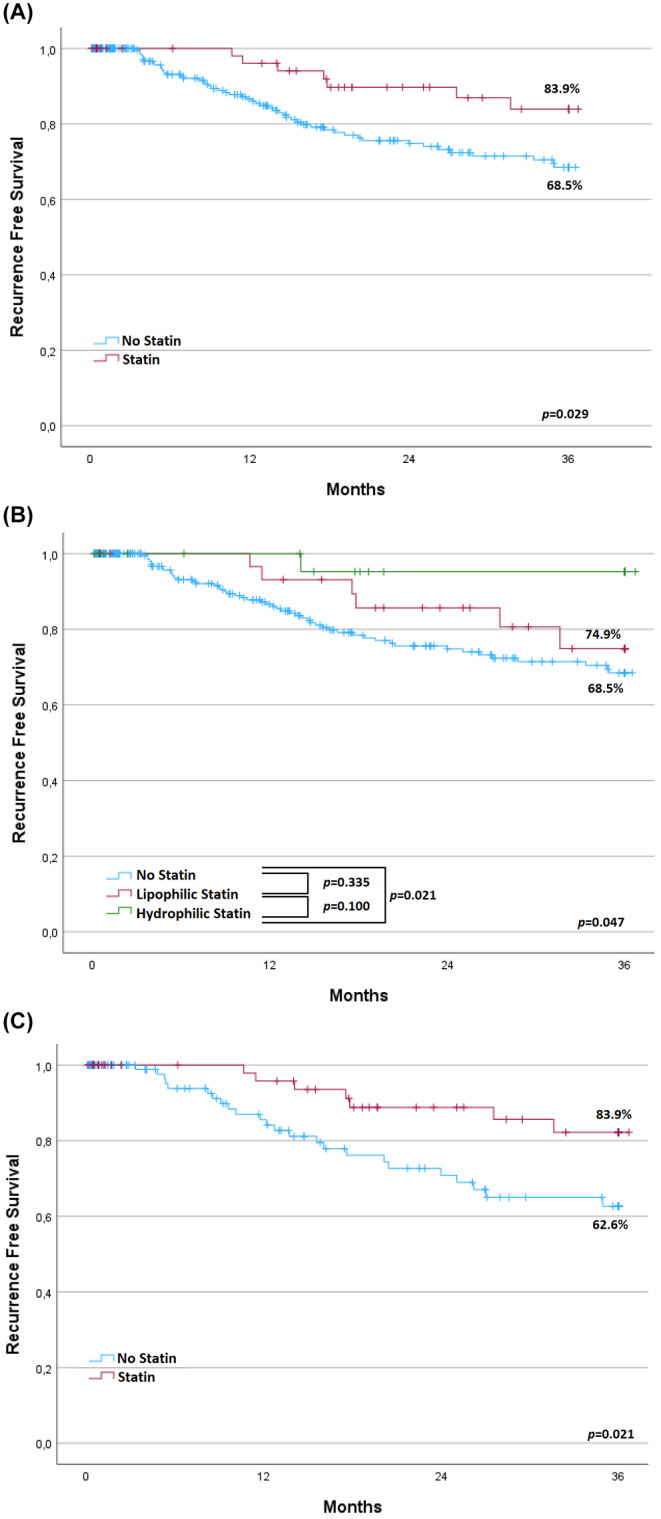
Kaplan–Meier curves comparing recurrence‐free survival between statin users and non‐statin users (A) in all patients, (B) according to the type of statins (lipophilic and hydrophilic), and (C) in a matched cohort for statins.

The characteristics of the statin users and non‐statin users are presented in Table S2. Patients in the statin group were older and the incidence of diabetes, hyperlipidemia, and cardiovascular diseases were higher in statin users. More patients in the statin group had multiple tumors than patients in the no statin group did (*p* = 0.033). Other baseline data and intra‐ and postoperative variables were not different between the two groups. After performing a 1:2 matching for statin use, 186 patients were included in the analysis (62 patients in statin group and 124 in the no statin group). As shown in Table S2, patient characteristics after matching were similar between the two groups, except for a higher incidence of hyperlipidemia in the statin group (*p* < 0.001). Survival analysis revealed a significantly higher RFS in the statin group before matching (three‐year RFS: 83.9% in statin group vs. 68.5% in no statin group; *p* = 0.029) (Figure 1A). The comparison of the RFS according to the type of statin (lipophilic and hydrophilic) revealed a higher RFS in patients using hydrophilic statins than in patients not using statins (*p* = 0.021), but no difference in RFS between patients using lipophilic statins and patients not using statins (*p* = 0.335) (Figure 1B). Evaluation of statin intensity showed that 10 patients used low intensity statins, 42 used moderate intensity statins, and 10 used high intensity statins. There were no significant differences in RFS between patients using low‐, moderate‐, or high intensity statins (Figure S2, *p* = 0.106). RFS analysis after matching confirmed the higher RFS in statin users than in non‐statin users (three‐year RFS: 83.9% vs. 62.6%, *p* = 0.021) (Figure 1C). Multivariable analysis in the matched cohort revealed that statin use (HR: 0.41; 95% CI, 0.17–0.97; *p* = 0.044) was an independent protective factor of recurrence after curative liver resection (Table 2). The assumption proportional hazards was not violated for statin (*p* = 0.266).

**TABLE 2:** Multivariate analysis of prognostic factors of recurrence free and overall survival in a matched cohort for statins.

	Recurrence free survival				Overall survival			
Variables	Univariate		Multivariate		Univariate		Multivariate	
	HR (95% CI)	*p*	HR (95% CI)	*p*	HR (95% CI)	*p*	HR (95% CI)	*p*
Male gender	1.12 (0.43–2.91)	0.822			26.68 (0.28–251.6)	0.347		
Age > 60 years	1.52 (0.46–5.00)	0.493			25.8 (0.19–349.2)	0.377		
BMI ≥30 kg/m^2^	0.76 (0.34–1.69)	0.502			0.82 (0.22–3.11)	0.775		
Diabetes mellitus	0.99 (0.49–2.01)	0.982			1.99 (0.58–6.81)	0.271		
HBV infection	0.62 (0.15–2.62)	0.521			1.86 (0.40–8.62)	0.427		
HCV infection	0.16 (0.02–1.17)	0.071	0.19 (0.3–1.44)	0.109	1.16 (0.25–5.40)	0.844		
Cirrhosis	0.51 (0.23–1.15)	0.105			1.33 (0.41–4.37)	0.636		
ASA class of 3–4	1.50 (0.69–3.26)	0.306			2.49 (0.66–9.40)	0.177		
AFP ≥200 ng/mL	0.61 (0.30–1.24)	0.176			1.24 (0.36–4.23)	0.735		
Platelet <150 /nL	0.75 (0.33–1.67)	0.476			4.55 (1.38–14.92)	**0.012**	6.43 (1.74–23.88)	**0.005**
Statin use	0.38 (0.16–0.89)	**0.027**	0.41 (0.17–0.97)	**0.044**	0.88 (0.26–3.02)	0.842	0.61 (0.16–2.26)	0.457
Metformin use	0.61 (0.25–1.47)	0.266			0.60 (0.13–2.78)	0.515		
Aspirin use	0.53 (0.22–1.29)	0.164			3.00 (0.91–9.85)	0.070	2.97 (0.82–10.65)	0.096
Major liver resection	1.15 (0.55–2.36)	0.714			1.46 (0.44–4.79)	0.532		
Blood loss >1000 mL	1.17 (0.48–2.84)	0.736			0.89 (0.11–7.31)	0.914		
Intraoperative blood transfusion	1.17 (0.36–3.87)	0.789			1.21 (0.15–9.54)	0.854		
Operative time > 150 min	0.82 (0.39–1.71)	0.596			085 (0.24–3.01)	0.797		
Multiple tumors	5.57 (2.36–13.17)	**<0.001**	6.34 (2.35–17.07)	**<0.001**	4.86 (1.28–18.44)	**0.020**	7.61 (1.74–33.36)	**0.007**
Bilobar tumor	1.48 (0.57–3.86)	0.420			1.62 (0.35–7.53)	0.535		
Tumor size ≥3 cm	1.86 (0.71–4.83)	0.206			0.38 (0.17–1.99)	0.387		
Tumor stage of T3–4	1.99 (0.94–4.23)	0.074	1.06 (0.45–2.48)	0.891	0.86 (0.17–3.98)	0.847		
High grade tumor (G3–4)	1.89 (0.88–3.97)	0.105			0.81 (0.17–3.74)	0.784		
Vascular invasion	2.81 (1.35–5.87)	**0.006**	2.65 (1.22–5.74)	**0.013**	0.78 (0.23–2.68)	0.701		

The bold values are the p values of those which are statistically significant.

No significant difference was seen in OS between the two groups (three‐year OS: 89.5% in statin users vs. 88.5% in non‐statin users; *p* = 0.730) before matching (Figure S3A). The OS of patients in the statin group was not different to that in the no statin group after propensity score matching (three‐year OS: 89.5% in statin users vs. 89.5% in non‐statin users; *p* = 0.842) (Figure S3B). The multivariable analysis of predictive factors of OS showed that statin use did not affect OS (Table 2). Comparing the recurrence/death survivals revealed a significant higher survival in statin users before (three‐year survival: 77.8% vs. 61.2%; *p* = 0.026) before and after matching (three‐year survival: 77.8% vs. 58.2%; *p* = 0.021) (Figure S4).

### 3.3 The effect of metformin use on survival outcomes

**FIGURE 2:**
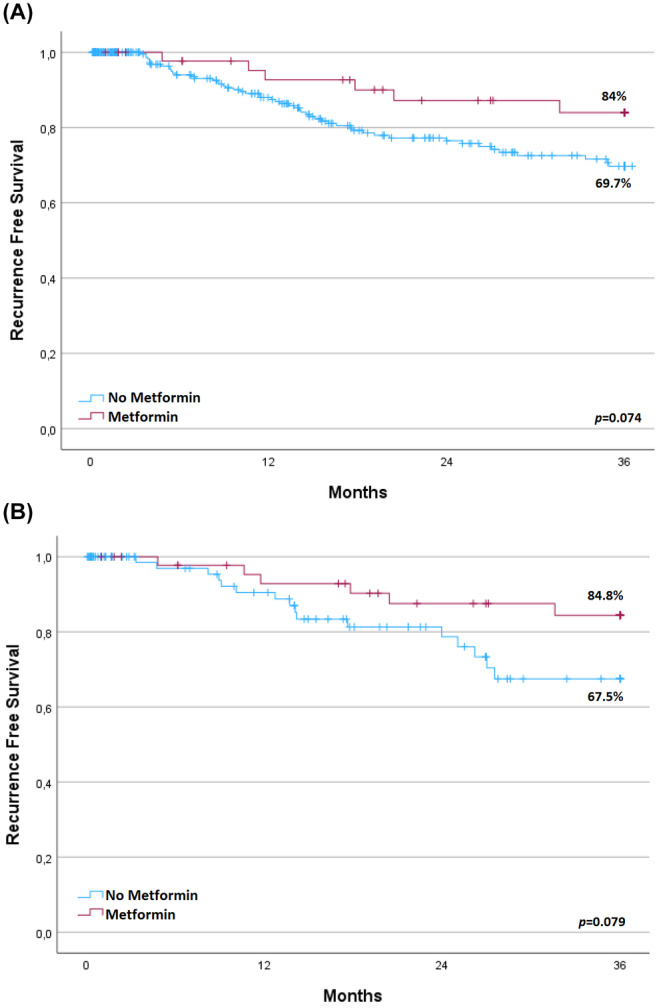
Kaplan–Meier curves comparing recurrence free survival between metformin users and non‐metformin users (A) in all patients and (B) in a matched cohort for metformin.

The characteristics of metformin users and non‐metformin users are presented in Table S3. The metformin group was older (*p* = 0.033) and had a higher proportion of males (*p* = 0.042) and a higher BMI (*p* = 0.033). All metformin users had diabetes while only 18.4% of non‐metformin users had diabetes (*p* = 0.001). The incidence of hyperlipidemia and cardiovascular diseases were also higher in metformin users. No other differences were observed between the two groups. Of the 305 patients who did not receive metformin, 94 were matched to the 47 metformin users. Except for the proportion of diabetes, the baseline characteristics and intraoperative and postoperative outcomes were similar between the matched groups (Table S3). Survival analysis before matching revealed no significant difference in RFS between the metformin and no metformin group (three‐year RFS: 84% in metformin users vs. 69.7% in non‐metformin users; *p* = 0.074) (Figure 2A). To evaluate the role of diabetes on HCC recurrence after curative liver resection, we compared the RFS between patients with and without diabetes, and found no significant difference (Figure S5, *p* = 0.120). Although statistically not significant, after propensity score matching, metformin users had a higher RFS than non‐metformin users did (three‐year RFS: 84.8% vs. 67.5%, *p* = 0.079) (Figure 2B). Multivariable analysis of patients in the matched cohort for metformin use did not reveal a significant protective effect against HCC recurrence after liver resection for using metformin (HR: 0.38; 95% CI: 0.15–1.01; *p* = 0.051, Table 3). The assumption proportional hazards was not violated for metformin (*p* = 0.804).

**TABLE 3:** Multivariable analysis of prognostic factors of recurrence free and overall survival in a matched cohort for metformin.

	Recurrence free survival				Overall survival			
Variables	Univariate		Multivariable		Univariate		Multivariable	
	HR (95% CI)	*p*	HR (95% CI)	*p*	HR (95% CI)	*p*	HR (95% CI)	*p*
Male gender	0.32 (0.17–0.86)	**0.024**	0.25 (0.08–0.71)	**0.010**	24.3 (0.04–133.3)	0.322		
Age > 60 years	0.86 (0.28–2.53)	0.779			1.24 (0.36–4.29)	0.735		
BMI ≥30 kg/m^2^	0.73 (0.27–1.98)	0.541			1.11 (0.39–3.16)	0.839		
Diabetes mellitus	0.89 (0.24–1.45)	0.248			3.01 (0.69–13.09)	0.142		
HBV infection	0.42 (0.06–3.15)	0.401			1.08 (0.25–4.70)	0.919		
HCV infection	0.04 (0.01–5.17)	0.192			1.20 (0.35–4.17)	0.766		
Cirrhosis	0.45 (0.17–1.15)	0.096	0.35 (0.13–0.95)	**0.038**	1.14 (0.45–2.89)	0.781		
ASA class of 3–4	0.57 (0.23–1.42)	0.231			2.91 (0.66–12.91)	0.159		
AFP ≥200 ng/mL	1.05 (0.45–2.46)	0.909			0.53 (0.21–1.34)	0.178		
Platelet <150 /nL	1.45 (0.49–4.29)	0.502			0.52 (0.19–1.39)	0.195		
Statin use	0.61 (0.22–1.66)	0.333			1.17 (0.44–3.12)	0.753		
Metformin use	0.44 (0.17–1.13)	**0.087**	0.38 (0.15–1.01)	**0.051**	0.61 (0.21–1.73)	0.354	0.55 (0.17–1.78)	0.315
Aspirin use	0.47 (0.16–1.40)	**0.178**			1.35 (0.64–4.25)	0.304		
Major liver resection	0.67 (0.25–1.81)	0.427			0.92 (0.33–2.58)	0.888		
Blood loss >1000 mL	0.69 (0.20–2.29)	0.550			2.50 (0.92–6.77)	**0.072**	1.64 (0.54–4.94)	0.379
Intraoperative blood transfusion	0.80 (0.18–3.48)	0.769			0.95 (0.22–4.19)	0.951		
Operative time > 150 min	0.71 (0.27–1.83)	0.476			2.95 (1.08–7.99)	**0.034**	2.99 (1.01–8.86)	**0.047**
Multiple tumors	1.19 (0.35–4.04)	0.776			2.26 (0.74–6.90)	0.153		
Bilobar tumor	1.35 (0.39–4.56)	0.531			1.42 (0.41–4.92)	0.581		
Tumor size ≥3 cm	2.16 (0.79–5.84)	**0.130**			1.11 (0.41–2.99)	0.833		
Tumor stage of T3–4	1.56 (0.65–3.73)	0.316			0.67 (0.22–2.05)	0.487		
High grade tumor (G3–4)	1.59 (0.64–3.85)	0.326			0.64 (0.18–2.22)	0.482		
Vascular invasion	2.59 (1.11–6.07)	**0.028**	2.48 (1.05–5.85)	**0.014**	3.15 (0.91–10.89)	**0.070**	3.05 (0.83–11.21)	0.092

The bold values are the p values of those which are statistically significant.

We also compared the RFS of metformin users and nonusers in a subgroup of patients with diabetes. Survival analysis in diabetic patients, revealed an RFS of 84% in metformin groups compared with 68.7% in no metformin group (Figure S6A, *p* = 0.071). After propensity score matching, metformin users had a statistically non‐significant longer RFS than patients with diabetes who did not receive metformin (84.1% vs. 60.8%, *p* = 0.069) (Figure S6B).

The OS was similar between metformin users and non‐metformin users (three‐year OS: 93.6% vs. 87.7%, *p* = 0.289) (Figure S7A), also after propensity score matching (three‐year OS: 93.6% vs. 79.7%, *p* = 0.059) (Figure S7B). Multivariable analysis revealed that metformin use did not affect OS (Table 3). Comparison of the recurrence/death free survival before matching revealed no significant difference between the metformin and no metformin group (three‐year survival: 75.6% in metformin users vs. 62.4% in non‐metformin users; *p* = 0.134) (Figure S8A). After propensity score matching, metformin users had a significantly higher recurrence/death free survival than non‐metformin users did (three‐year survival: 75.1% vs. 52.7%, *p* = 0.031) (Figure S8B).

### 3.4 The effect of aspirin use on survival outcomes

The characteristics of aspirin users and nonaspirin users are presented in Table S4. The aspirin group was older (*p* < 0.001), had a higher incidence of diabetes, hyperlipidemia, and cardiovascular disease (*p* for all <0.001) and cirrhosis (*p* = 0.043), and had higher ASA classes (*p* = 0.032). A higher proportion of nonaspirin users had multiple tumors and tumor grades of G 3–4 (*p* = 0.042 for both). Other variables were similar between the two groups. We performed a 2:1 propensity score matching analysis, in which 106 nonaspirin users were matched to 53 aspirin users. This analysis revealed a higher incidence of hyperlipidemia and cardiovascular diseases in aspirin users; all other patient characteristics and perioperative data were similar between the two groups (Table S4). The survival analysis of all patients showed a similar RFS between aspirin users and nonaspirin users (*p* = 0.385, Figure S9A), and this was also the case after matching (*p* = 0.361, Figure S9B). Multivariable analysis for RFS predictors in the matched cohort of aspirin users revealed no association between aspirin use and RFS after surgery. The assumption proportional hazards was not violated for aspirin (*p* = 0.896).

The Kaplan–Meier survival analysis showed no differences in OS between aspirin users and nonaspirin users before (*p* = 0.205, Figure S10A) and after propensity score matching (*p* = 0.090, Figure S10B). Multivariable analysis of OS showed that aspirin use did not affect mortality after liver resection (Table 4). No differences were seen in recurrence/death free survival between aspirin users and nonaspirin users before (*p* = 0.414, Figure S11A) and after propensity score matching (*p* = 0.210, Figure S11B).

**TABLE 4:** Multivariable analysis of prognostic factors of recurrence free and overall survival in a matched cohort for aspirin.

	Recurrence free survival				Overall survival			
Variables	Univariate		Multivariable		Univariate		Multivariable	
	HR (95% CI)	*p*	HR (95% CI)	*p*	HR (95% CI)	*p*	HR (95% CI)	*p*
Male gender	1.67 (0.39–7.03)	0.483			23.3 (0.02–235.9)	0.510		
Age > 60 years	1.66 (0.39–6.96)	0.491			1.06 (0.14–8.41)	0.618		
BMI ≥30 kg/m^2^	0.61 (0.26–1.41)	0.247			0.86 (0.22–3.36)	0.829		
Diabetes mellitus	0.71 (0.35–1.46)	0.352			3.21 (0.68–15.11)	0.140		
HBV infection	0.83 (0.25–2.75)	0.760			1.89 (0.40–8.91)	0.442		
HCV infection	0.31 (0.07–1.31)	0.112			1.21 (0.26–5.68)	0.812		
Cirrhosis	0.82 (0.39–1.74)	0.596			0.88 (0.25–3.15)	0.853		
ASA class of 3–4	0.81 (0.33–1.97)	0.643			2.71 (0.33–22.08)	0.350		
AFP ≥200 ng/mL	2.21 (1.07–4.58)	**0.031**	2.35 (1.13–4.89)	**0.023**	1.01 (0.28–3.57)	0.990		
Platelet <150 /nL	0.74 (0.34–1.61)	0.446			2.12 (0.59–7.53)	0.245		
Statin use	0.46 (0.19–1.08)	**0.075**	0.45 (0.18–1.09)	0.078	1.11 (0.31–3.93)	0.874		
Metformin use	0.45 (0.16–1.33)	0.148			0.56 (0.12–2.64)	0.463		
Aspirin use	0.65 (0.29–1.47)	0.304	0.91 (0.38–2.17)	0.838	2.85 (0.80–10.12)	0.105	2.38 (0.66–8.63)	0.188
Major liver resection	1.47 (0.71–3.06)	0.301			1.34 (0.38–4.75)	0.650		
Blood loss >1000 mL	1.63 (0.69–3.84)	0.263			2.85 (0.68–11.96)	0.151		
Intraoperative blood transfusion	0.59 (0.14–2.53)	0.485			1.38 (0.17–11.36)	0.764		
Operative time > 150 min	0.98 (0.47–2.03)	0.964			2.35 (0.58–9.43)	0.226		
Multiple tumors	2.51 (0.87–7.26)	**0.089**	3.33 (1.07–10.34)	**0.037**	5.73 (1.47–22.25)	**0.012**	4.74 (1.19–18.84)	**0.027**
Bilobar tumor	1.58 (0.55–4.54)	0.394			2.36 (0.50–11.15)	0.276		
Tumor size ≥3 cm	1.25 (0.58–2.67)	0.567			0.85 (0.24–3.02)	0.804		
Tumor stage of T3–4	1.77 (0.81–3.88)	0.154			1.77 (0.45–6.88)	0.410		
High grade tumor (G3–4)	1.46 (0.67–3.20)	0.338			1.35 (0.35–5.23)	0.664		
Vascular invasion	1.97 (0.96–4.04)	**0.065**	5.35 (1.18–24.13)	**0.029**	1.09 (0.31–3.86)	0.894		

The bold values are the p values of those which are statistically significant.

## 4 DISCUSSION

The results of the present study revealed that perioperative use of statin and metformin is associated with a reduced risk of HCC recurrence after surgery but has no impact on OS of the patients. The protective effect of statins was higher in patients using hydrophilic statins and was not dose dependent. To our knowledge, this is the first study to evaluate the impact of these medications on HCC recurrence after liver resection in a Western population. Previous studies have focused on Asian‐Pacific populations, which have different demographics and different primary causes of HCC. For example, HBV‐related HCC is more frequent in the Asian population, whereas HCV‐ and alcohol‐related HCC are more frequent in Western countries.^12^

Several studies have demonstrated that statins can prevent cancer, particularly HCC, via their anti‐inflammatory and antioncogenic effects.^13^, ^14^, ^15^, ^16^ Statins can also reduce the risk of HCC in patients with diabetes,^17^ as well as in patients with HBV and HCV.^18^ Some experimental studies have reported that statins can improve liver regeneration and angiogenesis after massive liver resections through anti‐inflammatory mechanisms.^19^, ^20^ In a preliminary trial on 20 patients, statin therapy before liver resection decreased the risk of ischemia reperfusion injury.^21^ Recent studies from Asian‐Pacific regions have shown that statins prevent HCC recurrence after liver resection.^7^, ^22^, ^23^, ^24^, ^25^ Our present findings in a population of European patients are in line with those of Nishio et al.^25^ and Kawaguchi et al.,^24^ who showed that statins significantly increased RFS but not OS.

How the type and dose of statins affect HCC recurrence after liver resection has not been evaluated previously. We found that hydrophilic statins increased the RFS of HCC patients after resection while lipophilic statins did not. This contradicts previous findings of some studies that lipophilic statins have more anticancer activity than hydrophilic statins.^26^, ^27^ However, in agreement with our findings, this anticancer effect of lipophilic statins was not confirmed for HCC,^28^ possibly because hydrophilic statins are more hepatoselective than lipophilic statins. The mechanism underlying the protective effect of statins against HCC recurrence has not been completely elucidated, and may be different to those of non‐liver cancers. Possible anticancer mechanisms of statins include activating the tumor apoptosis cascade,^29^ regulating the mevalonate pathway by inhibiting downstream products,^30^ and inducing proteasomal degradation and autophagy.^31^, ^32^

The effect of diabetes mellitus on the survival of patients with HCC is controversial.^33^ The present study found no effect of diabetes on survival after hepatic resection in patients with HCC. In contrast to our findings, some studies have demonstrated an increased risk of recurrence and mortality in patients with HCC who also have diabetes.^34^, ^35^ In agreement with our findings, other studies have shown no association between diabetes and survival of patients with HCC after resection.^36^ These controversial findings may be related to the effects of the diabetes medication metformin.

Recent studies have shown that metformin has a preventive effect against HCC in patients with newly diagnosed type 2 diabetes^33^, ^37^ and also has a chemoprotective effects in patients with HCC and affects the survival of these patients.^38^ However, the preventive effect of metformin on recurrence and mortality in HCC patients after resection remains controversial. Some studies found no association between metformin and survival outcomes in HCC patients after resection.^35^, ^39^ Other studies have reported a favorable effect of metformin on RFS and OS.^40^, ^41^, ^42^, ^43^ Our analysis on matched patients group with diabetes showed that metformin reduces the HCC recurrence rate after liver resection (RFS: 84.1% vs. 60.8%), but the difference was not statistically significant. However, due to the small number of patients with diabetes who did not receive metformin, our analysis lacks enough power and further studies on patients with diabetes are needed.

The antitumor mechanism of metformin in HCC has not been completely elucidated. A systematic review found that metformin can potentially inhibit HCC growth.^44^ Several studies have shown that metformin inhibits cancer cell growth in patients with HCC by activating the dependent AMP‐activated protein kinase pathway and by inducing apoptosis and cell arrest by activating the independent AMP‐activated protein kinase pathway.^45^ Metformin can also reduce cancer cell proliferation by inhibiting mTOR protein synthesis.^46^ It also increases glucose uptake and reduces insulin levels via the liver gluconeogenesis cycle, which can decrease cancer cell proliferation.^47^

The antitumor mechanism of aspirin in HCC is also unclear. Aspirin prevents the development of various cancers, including liver cancer.^48^, ^49^, ^50^, ^51^ Some studies have showed that aspirin can improve RFS in patients with HCC after resection.^9^, ^39^ Data from the national database of HCC patients in Taiwan^9^ showed that aspirin reduces the risk of early HCC recurrence after liver resection. Another study using the same database showed that aspirin and clopidogrel increase the RFS and OS of patients with HBV‐related HCC after liver resection.^39^ However, a meta‐analysis on HCC recurrence after liver resection showed that aspirin alone does not reduce recurrence.^52^ The present study adds to these findings by showing no association between aspirin use and survival outcomes after curative liver resection for HCC.

This study has some limitations. It was not a randomized controlled trial, therefore selection bias could have influenced the outcomes. To reduce the impact of selection and confounding biases, we performed a propensity score matching analysis; however, there are still some limitation to our findings due to potentially unmeasured confounders. Another limitation is that we did not evaluate the adverse effects of the investigated drugs. Furthermore, we did not determine the dose–response relationship between statin, metformin, and aspirin use and survival outcomes. Finally, we did not assess the cumulative effect of other medications and blood glucose levels, which may have affected the survival outcomes.

In conclusion, our results suggest that perioperative use of statins, but not metformin and aspirin, can protect against recurrence after liver resection in patients with HCC. Metformin may improve RFS in patients with diabetes. However, future prospective controlled trials are needed to investigate the impact of these medications on survival after liver resection in patients with HCC, and to examine the mechanisms underlying their protective effect.

## AUTHOR CONTRIBUTIONS

**Elias Khajeh:** Conceptualization (equal); data curation (equal); formal analysis (lead); methodology (lead); writing – original draft (equal). **Ehsan Aminizadeh:** Data curation (equal); formal analysis (equal); methodology (supporting); writing – original draft (equal). **Arash Dooghaie Moghadam:** Conceptualization (supporting); data curation (supporting); writing – original draft (supporting). **Ali Ramouz:** Data curation (supporting); formal analysis (supporting); methodology (supporting); writing – original draft (supporting). **Rosa Klotz:** Methodology (supporting); writing – review and editing (equal). **Mohammad Golriz:** Data curation (supporting); supervision (supporting); writing – review and editing (equal). **Uta Merle:** Methodology (supporting); supervision (supporting); writing – review and editing (equal). **Christoph Springfeld:** Conceptualization (supporting); methodology (supporting); supervision (supporting); writing – review and editing (equal). **De‐Hua Chang:** Methodology (supporting); supervision (supporting); writing – review and editing (equal). **Thomas Longerich:** Methodology (supporting); supervision (supporting); writing – review and editing (equal). **Markus W. Büchler:** Conceptualization (supporting); supervision (equal); writing – review and editing (equal). **Arianeb Mehrabi:** Conceptualization (lead); formal analysis (supporting); methodology (equal); supervision (lead); writing – original draft (supporting); writing – review and editing (lead).

## FUNDING INFORMATION

None.

## CONFLICT OF INTEREST STATEMENT

The authors declare no conflict of interest.

## ETHICS STATEMENT

The independent ethics committee of Heidelberg University approved the study protocol (S‐754/2018).

## CONSENT

All patients signed an informed consent form at admission, allowing their data to be used for study purposes in the future.

## Supporting information


**Data S1.** Supporting Information.

## Data Availability

The data used for this study can be made available upon request to the corresponding author and after agreement of the ethics committee of the Heidelberg University.
