# Supplementary material for: Association of perioperative use of statins, metformin, and aspirin with recurrence after curative liver resection in patients with hepatocellular carcinoma: A propensity score matching analysis
Source: Cancer Med. 2023 Sep 22;12(19):19548–59. doi: 10.1002/cam4.6569 (PMC10587989; doi:10.1002/cam4.6569)

Supplementary Materials

Association of Perioperative Use of Statins, Metformin, and Aspirin with Recurrence after Curative Liver Resection in Patients with Hepatocellular Carcinoma: A Propensity Score Matching Analysis

Elias Khajeh[1], Ehsan Aminizadeh[1], Arash Dooghaie Moghadam[1], Ali Ramouz[1], Rosa Klotz[1], Mohammad Golriz[1, 2], Uta Merle[2, 3], Christoph Springfeld[2, 4], De-Hua Chang[2, 5], Thomas Longerich[2, 6], Markus W. Büchler[1], Arianeb Mehrabi[1,2]

[1]Department of General, Visceral and Transplantation Surgery, Heidelberg University Hospital, Heidelberg, Germany

[2]Liver Cancer Center Heidelberg (LCCH), Heidelberg University Hospital, Heidelberg, Germany

[3]Department of Internal Medicine IV, Gastroenterology & Hepatology, Heidelberg University Hospital, Heidelberg, Germany

[4]National Center for Tumor Diseases, Department of Medical Oncology, Heidelberg University Hospital, Heidelberg, Germany

[5]Department of Diagnostic and Interventional Radiology, Heidelberg University Hospital, Heidelberg, Germany

[6]Institute of Pathology, Heidelberg University Hospital, Heidelberg, Germany

Correspondence:

Professor Arianeb Mehrabi, MD, FICS, FEBS, FACS
Head of the Division of Liver Surgery and Abdominal Transplantation
Department of General, Visceral and Transplantation Surgery
Heidelberg University Hospital, Germany
Im Neuenheimer Feld 420, 69120, Heidelberg, Germany
E-mail: Arianeb.Mehrabi@med.uni-heidelberg.de

Supplementary Tables

Table S1. Univariate and multivariate analyses of factors associated with 5-year recurrence free and overall survival after
curative liver resection in patients with HCC.

	**Recurrence free survival**				**Overall survival**			
**Variables**	**Univariate**		**Multivariate**		**Univariate**		**Multivariate**	
	**HR (95% CI)**	***p***	**HR (95% CI)**	***p***	**HR (95% CI)**	***p***	**HR (95% CI)**	***p***
Male gender	0.82 (0.45 – 1.47)	0.503			6.46 (0.87 – 47.91)	0.068	5.93 (0.79 –44.3)	0.083
Age >60 years	0.98 (0.56 – 1.73)	0.954			1.54 (0.57 – 4.15)	0.392		
BMI ≥30 kg/m^2	0.89 (0.48 – 1.62)	0.701			0.87 (0.30 – 2.17)	0.672		
Diabetes mellitus	0.68 (0.38 – 1.24)	0.210			1.17 (0.49 – 2.76)	0.720		
HBV infection	0.55 (0.22 – 1.37)	0.198			1.63 (0.61 – 4.39)	0.334		
HCV infection	0.39 (0.15 – 0.97)	**0.043**	0.67 (0.25 – 1.81)	0.438	0.93 (0.32 – 2.75)	0.903		
Cirrhosis	0.52 (0.38 – 0.99)	**0.049**	0.65 (0.35 – 1.19)	0.165	0.83 (0.36 – 1.90)	0.669		
ASA class of 3-4	0.60 (0.33 – 1.10)	0.110			1.31 (0.49 – 3.46)	0.589		
AFP ≥ 200 ng/ml	0.80 (0.48 – 1.34)	0.407			1.99 (0.82 – 4.85)	0.128		
Platelet <150 /nl	1.10 (0.63 – 1.93)	0.720			1.34 (0.57 –3.17)	0.502		
Statin use	0.43 (0.19 – 0.95)	**0.037**	0.42 (0.19 – 0.94)	**0.036**	0.83 (0.28 – 2.43)	0.731		
Metformin use	0.43 (0.18 – 0.99)	0.050	0.56 (0.23 – 1.34)	0.192	0.46 (0.11 – 1.98)	0.301		
Aspirin use	0.73 (0.35 – 1.55)	0.416			1.81 (0.71 – 4.59)	0.211		
Major liver resection	1.52 (0.90 – 2.55)	0.116			1.75 (0.77 – 3.97)	0.181		
Blood loss >1000 ml	1.20 (0.66 – 2.21)	0.549			2.07 (0.86 – 5.01)	0.105		
Intraoperative blood transfusion	0.91 (0.36 – 2.29)	0.848			1.59 (0.46 – 5.43)	0.458		
Operative time >150 min	1.19 (0.71 – 2.01)	0.504			3.01 (1.22 – 7.41)	**0.016**	3.05 (1.21 – 7.69)	**0.018**
Multiple tumors	1.69 (0.95 – 3.02)	0.072	1.67 (0.91 – 3.04)	0.095	3.51 (1.52 – 8.14)	**0.003**	3.49 (1.47 – 8.28)	**0.005**
Bilobar tumor	1.31 (0.64 – 2.66)	0.460			1.45 (0.49 – 4.29)	0.494		
Tumor size ≥ 3 cm	2.54 (1.32 – 4.91)	**0.005**	2.25 (1.14 – 4.48)	**0.020**	1.14 (0.46 –2.80)	0.772		
Tumor stage of T3-4	1.81 (1.01 – 3.22)	**0.044**	1.54 (0.86 – 2.75)	0.150	1.72 (0.67 – 4.39)	0.259		
High grade tumor (G3-4)	1.71 (0.97 – 3.02)	0.062	1.39 (0.78 – 2.46)	0.260	0.59 (0.17 – 2.00)	0.401		
Vascular invasion	3.21 (1.89 – 5.44)	**<0.001**	2.46 (1.42 – 4.26)	**0.001**	2.59 (0.88 – 7.61)	0.084	2.51 (0.82 – 7.62)	0.104

Table S2. Baseline characteristics and intraoperative and postoperative outcomes of the patients in the statin group and no statin group (unmatched and matched).

Variables	Before matching		*p*	After matching		*p*
	**Statin (n = 62)**	**No statin (n = 291)**		**Statin (n = 62)**	**No statin (n=124)**	
Preoperative data						
Gender (male), n (%)	53 (85.5)	224 (77)	0.144	53 (85.5)	104 (83.9)	0.775
Age, years, mean ± SD	69.3 ± 7.7	62.7 ± 11.4	**0.001**	69.3 ± 7.7	67.9 ± 8.3	0.279
BMI ≥30 kg/m^2, n (%)	20 (32.3)	64 (22.1)	0.092	20 (32.3)	35 (28.2)	0.570
Diabetes mellitus, n (%)	32 (51.6)	71 (24.4)	**0.001**	32 (51.6)	50 (40.3)	0.144
Hyperlipidemia, n (%)	62 (100)	95 (32.6)	**<0.001**	62 (100)	34 (35.4)	**<0.001**
Cardiovascular disease, n (%)	43 (69.4)	122 (41.9)	**<0.001**	43 (69.4)	88 (71)	0.486
HBV infection, n (%)	6 (9.7)	38 (13.1)	0.458	6 (9.7)	11 (8.9)	0.857
HCV infection, n (%)	9 (14.5)	46 (15.8)	0.794	9 (14.5)	15 (12.1)	0.643
Cirrhosis, n (%)	23 (37.1)	141 (48.5)	0.103	23 (37.1)	49 (39.5)	0.749
Child-Pugh classification			0.588			0.437
A, n (%)	23 (100)	135 (95.7)		23 (100)	48 (38.7)	
B, n (%)	0 (0)	6 (4.3)		0 (0)	1 (0.8)	
ASA score 3-4, n (%)	27 (43.6)	122 (41.8)	0.319	27 (43.6)	61 (66.3)	0.103
AFP ≥200 ng/ml, n (%)	33 (53.2)	151 (51.9)	0.862	33 (53.2)	72 (58.1)	0.530
Platelet <150/nl, n (%)	15 (24.2)	85 (29.2)	0.423	15 (24.2)	27 (21.8)	0.710
Intraoperative data						
Major liver resection, n (%)	23 (37.1)	115 (39.5)	0.719	23 (37.1)	49 (39.5)	0.749
Blood loss, ml, mean ± SD	734 ± 650	772 ± 775	0.779	734 ± 650	665 ± 569	0.475
Intraoperative blood transfusion, n (%)	10 (18.2)	31 (11.8)	0.198	10 (18.2)	11 (9.7)	0.120
Operation time, min, mean ± SD	162 ± 68	163 ± 69	0.942	162 ± 68	163 ± 60	0.925
Postoperative data						
Multiple tumors, n (%)	6 (9.7)	62 (21.5)	**0.033**	6 (9.7)	10 (8.1)	0.712
Bilobar tumors, n (%)	9 (14.5)	44 (15.1)	0.904	9 (14.5)	17 (13.7)	0.881
Maximum tumor size >3 cm, n (%)	50 (80.6)	202 (71.4)	0.089	50 (80.6)	100 (80.6)	1
Tumor stage of T3-T4, n (%)	14 (23.7)	49 (18.2)	0.332	14 (23.7)	25 (20.3)	0.600
High tumor grade (G3-4), n (%)	9 (14.5)	48 (17.6)	0.549	9 (14.5)	19 (15.3)	0.885
Major complications (Clavien-Dindo≥3), n (%)	7 (11.3)	37 (12.7)	0.737	7 (11.3)	16 (12.9)	0.944
PHLF, n (%)	2 (3.2)	8 (2.7)	0.692	2 (3.2)	4 (3.2)	1
PHBL, n (%)	2 (3.2)	13 (4.5)	1	2 (3.2)	8 (6.5)	0.500
PHH, n (%)	2 (3.2)	8 (2.7)	0.692	2 (3.2)	3 (2.4)	1

Table S3. Characteristics, intraoperative outcomes, and postoperative outcomes of the patients in the metformin group and no metformin group (unmatched and matched).

Variables	Before matching		*p*	After matching		*p*
	**Metformin (n = 48)**	**No metformin (n = 305)**		**Metformin (n = 47)**	**No metformin (n = 94)**	
Preoperative data						
Gender (male), n (%)	43 (89.6)	234 (76.7)	**0.042**	42 (89)	83 (88.3)	0.851
Age, years, mean ± SD	67.4 ± 6.5	63.3 ± 11.6	**0.033**	67.4 ± 6.5	65.5 ± 13.8	0.380
BMI ≥30 kg/m^2, n (%)	15 (31.2)	53 (17.3)	**0.033**	15 (31.9)	22 (23.4)	0.180
Diabetes mellitus, n (%)	48 (100)	56 (18.4)	**0.001**	47 (100)	53 (56.4)	**0.001**
Hyperlipidemia, n (%)	29 (60.4)	128 (42)	**0.017**	28 (59.6)	53 (56.4)	0.074
Cardiovascular disease, n (%)	32 (66.7)	133 (43.6)	**0.003**	32 (68)	51 (54.3)	0.104
HBV infection, n (%)	3 (6.3)	41 (13.4)	0.158	3 (6.4)	10 (10.6)	0.544
HCV infection, n (%)	6 (12.5)	49 (16.1)	0.522	6 (12.8)	12 (12.8)	1
Cirrhosis, n (%)	22 (45.8)	142 (46.6)	0.923	21 (44.7)	39 (41.5)	0.718
Child-Pugh classification			1			0.976
A, n (%)	22 (50)	136 (95.8)		21 (44.7)	37 (39.3)	
B, n (%)	0 (0)	6 (4.2)		0 (0)	2 (2.1)	
ASA score 3-4, n (%)	22 (45.8)	132 (43.3)	0.332	21 (44.6)	46 (48.9)	0.645
AFP ≥200 ng/ml, n (%)	36 (75)	212 (69.5)	0.428	36 (77)	58 (61.7)	0.055
Platelet <150/nl, n (%)	9 (18.7)	91 (29.8)	0.113	9 (19.1)	22 (23.4)	0.383
Intraoperative data						
Major liver resection, n (%)	23 (47)	115 (37.7)	0.522	23 (48)	50 (53.1)	0.797
Blood loss, ml, mean ± SD	632 ± 767	787 ± 751	0.051	580 ± 553	728 ± 658	0.138
Intraoperative blood transfusion, n (%)	6 (13.6)	35 (12.8)	0.874	6 (13.6)	14 (14.8)	0.513
Operation time, min, mean ± SD	149 ± 58	165 ± 71	0.227	149 ± 58	160 ± 78	0.382
Postoperative data						
Multiple tumors, n (%)	5 (10.4)	63 (20.9)	0.084	5 (10.6)	12 (10.6)	0.715
Bilobar tumors, n (%)	3 (6.3)	50 (16.41)	0.062	3 (6.4)	17 (18.1)	0.061
Maximum tumor size >3 cm, n (%)	33 (68.7)	219 (71.4)	0.579	32 (68.1)	68 (68)	0.600
Tumor stage of T3-T4, n (%)	12 (26.1)	51 (18.1)	0.331	12 (25.5)	32 (34.4)	0.321
High tumor grade (G3-4), n (%)	9 (18.7)	45 (16)	0.614	9 (19.1)	18 (19.1)	1
Major complications (Clavien-Dindo≥3), n (%)	4 (8.3)	40 (13.1)	0.763	4 (8.5)	16 (17)	0.771
PHLF, n (%)	2 (4.2)	8 (2.6)	0.628	2 (4.3)	5 (5.3)	1
PHBL, n (%)	1 (2.1)	14 (4.6)	0.703	1 (2.1)	7 (7.4)	0.269
PHH, n (%)	0 (0)	10 (3.3)	0.356	0 (0)	2 (2.1)	0.553

Table S4. Characteristics, intraoperative outcomes, and postoperative outcomes of the patients in aspirin group and no aspirin group (unmatched and matched).

Variables	Before Matching		*p*	After Matching		*p*
	**Aspirin (n = 53)**	**No Aspirin (n = 300)**		**Aspirin (n = 53)**	**No Aspirin (n = 106)**	
Preoperative data						
Gender (male), n (%)	47 (88.7)	230 (76.7)	0.051	47 (88.7)	96 (90.6)	0.708
Age, years, mean ± SD	68.6 ± 5.9	63.1 ± 11.6	**<0.001**	68.6 ± 5.9	68.9 ± 8.3	0.802
BMI ≥30 kg/m², n (%)	16 (34)	57 (25.4)	0.348	16 (34)	31 (29)	0.902
Diabetes mellitus, n (%)	32 (60.4)	71 (23.7)	**<0.001**	32 (60.4)	47 (44.3)	0.057
Hyperlipidemia, n (%)	37 (69.8)	120 (40)	**<0.001**	37 (69.8)	58 (54.8)	**0.017**
Cardiovascular disease, n (%)	53 (100)	112 (37.3)	**<0.001**	53 (100)	69 (65.8)	**0.006**
HBV infection, n (%)	3 (5.7)	41 (13.7)	0.112	3 (5.7)	13 (12.3)	0.192
HCV infection, n (%)	7 (13.2)	48 (16)	0.613	7 (13.2)	15 (14.2)	0.871
Cirrhosis, n (%)	18 (34)	146 (48.7)	**0.043**	18 (34)	46 (43.4)	0.253
Child-Pugh classification			0.379			1
A, n (%)	18 (34)	140 (46.7)		18 (34)	35 (42.4)	
B, n (%)	0 (0)	6 (2%)		0 (0)	1 (0.9)	
ASA score 3-4, n (%)	30 (56.6)	124 (41.3)	**0.032**	30 (56.6)	52 (49)	0.188
AFP ≥200 ng/ml, n (%)	39 (73.6)	209 (69.7)	0.558	39 (73.6)	58 (54.7)	0.256
Platelet <150/nl, n (%)	14 (26.4)	86 (28.7)	0.744	14 (26.4)	23 (21.7)	0.507
Intraoperative data						
Major liver resection, n (%)	18 (34)	120 (40)	0.413	18 (34)	38 (35.8)	0.814
Blood loss, ml, mean ± SD	695 ± 713	777 ± 762	0.283	695 ± 713	639 ± 510	0.588
Intraoperative blood transfusion, n (%)	6 (12.8)	35 (12.9)	0.978	6 (12.8)	13 (13.5)	0.898
Operation time, min, mean ± SD	154 ± 57	164 ± 71	0.659	154 ± 57	168 ± 73	0.703
Postoperative data						
Multiple tumors, n (%)	5 (9.4)	63 (21.2)	**0.042**	5 (9.4)	9 (8.5)	1
Bilobar tumors, n (%)	5 (9.4)	48 (16)	0.219	5 (9.4)	14 (13.2)	0.489
Maximum tumor size >3 cm, n (%)	36 (67.9)	216 (72.7)	0.472	36 (67.9)	77 (72.6)	0.536
Tumor stage of T3-T4, n (%)	12 (23.5)	51 (18.4)	0.389	12 (23.5)	18 (17)	0.762
High tumor grade (G3-4), n (%)	6 (11.3)	48 (16)	**0.042**	6 (11.3)	13 (12.3)	0.864
Major complications (Clavien-Dindo≥3), n (%)	6 (11.3.)	38 (12.7)	1	6 (11.3)	13 (12.2)	1
PHLF, n (%)	2 (3.8)	8 (2.7)	0.654	2 (3.8)	3 (2.8)	1
PHBL, n (%)	3 (5.7)	12 (4)	0.482	3 (5.7)	4 (3.8)	0.687
PHH, n (%)	2 (3.8)	8 (2.7)	0.648	2 (3.8)	2 (1.9)	0.613

Supplementary Figures

Figure S1. Consort flow diagram of the study.

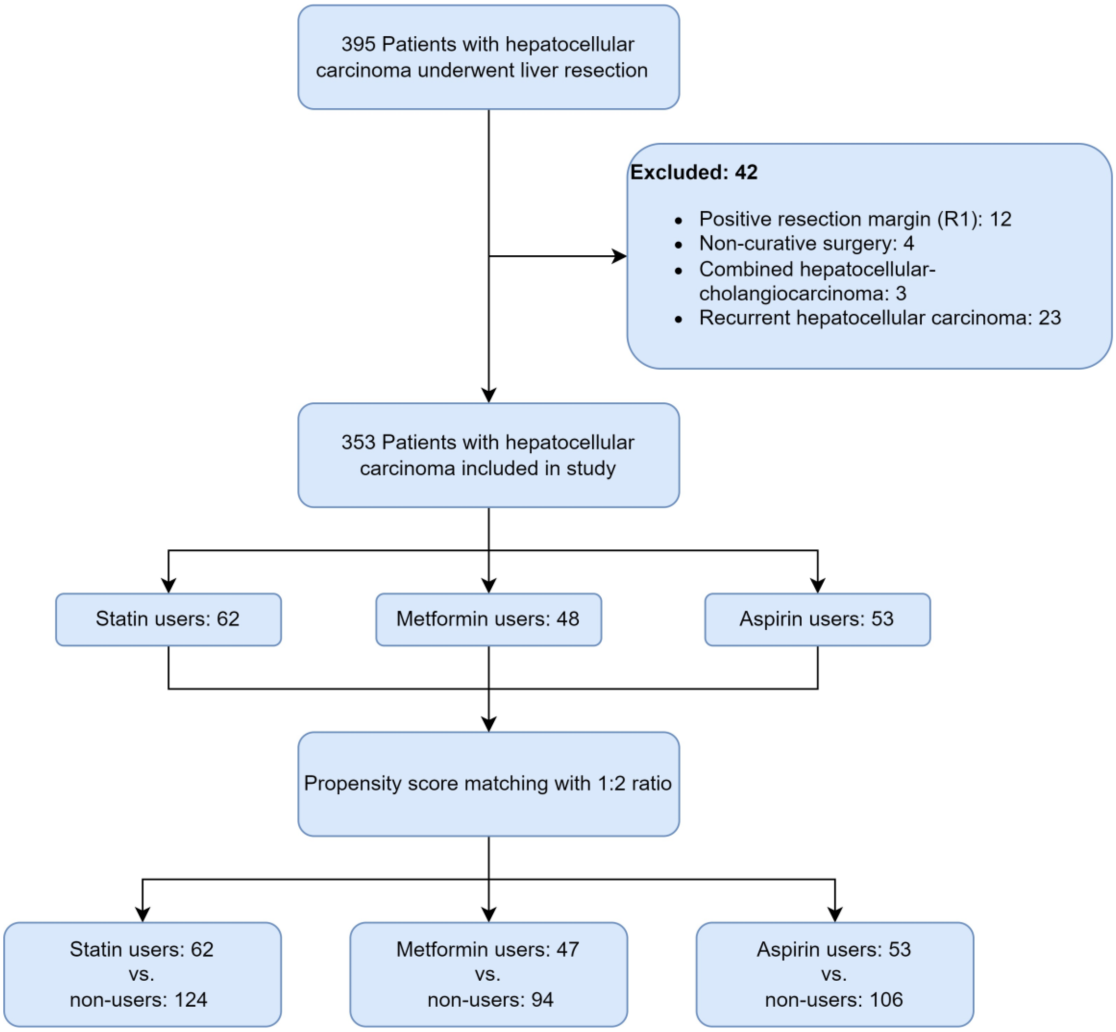

Figure S2. Kaplan–Meier curve comparing recurrence free survival in statin users according to the intensity of the used statins.

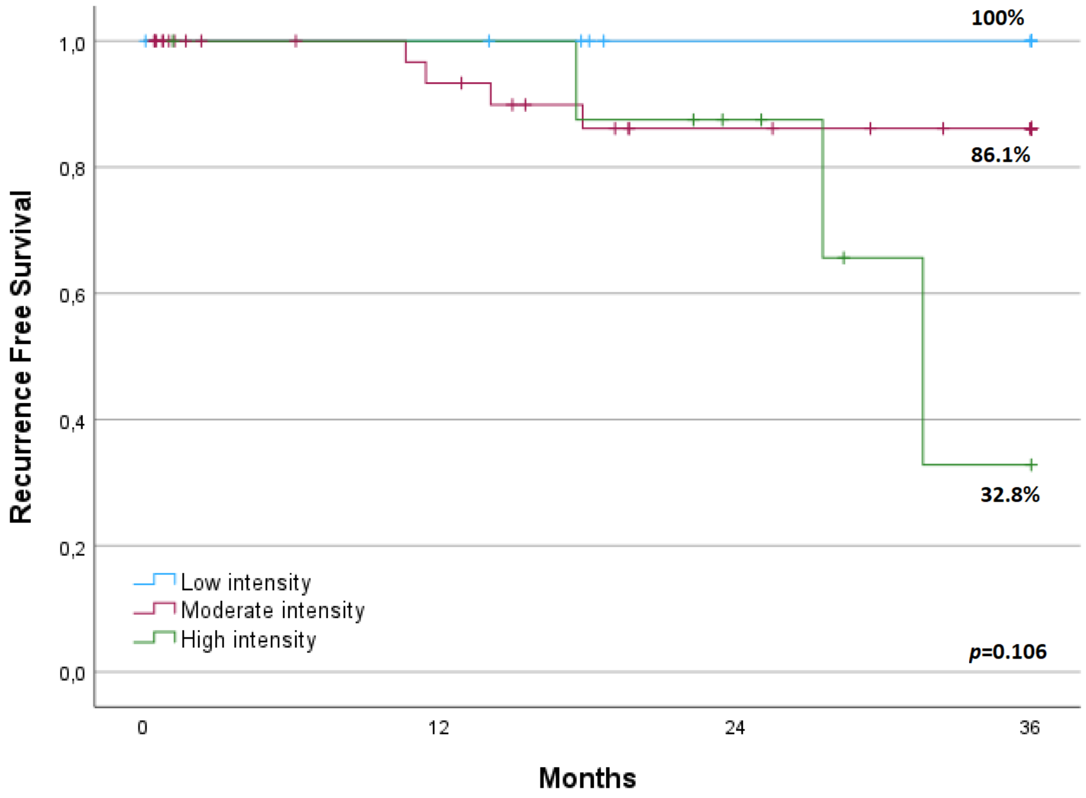

Figure S3. Kaplan–Meier curves comparing cumulative overall survival between statin users and non-statin users A) in all patients and B) in a matched cohort for statins.

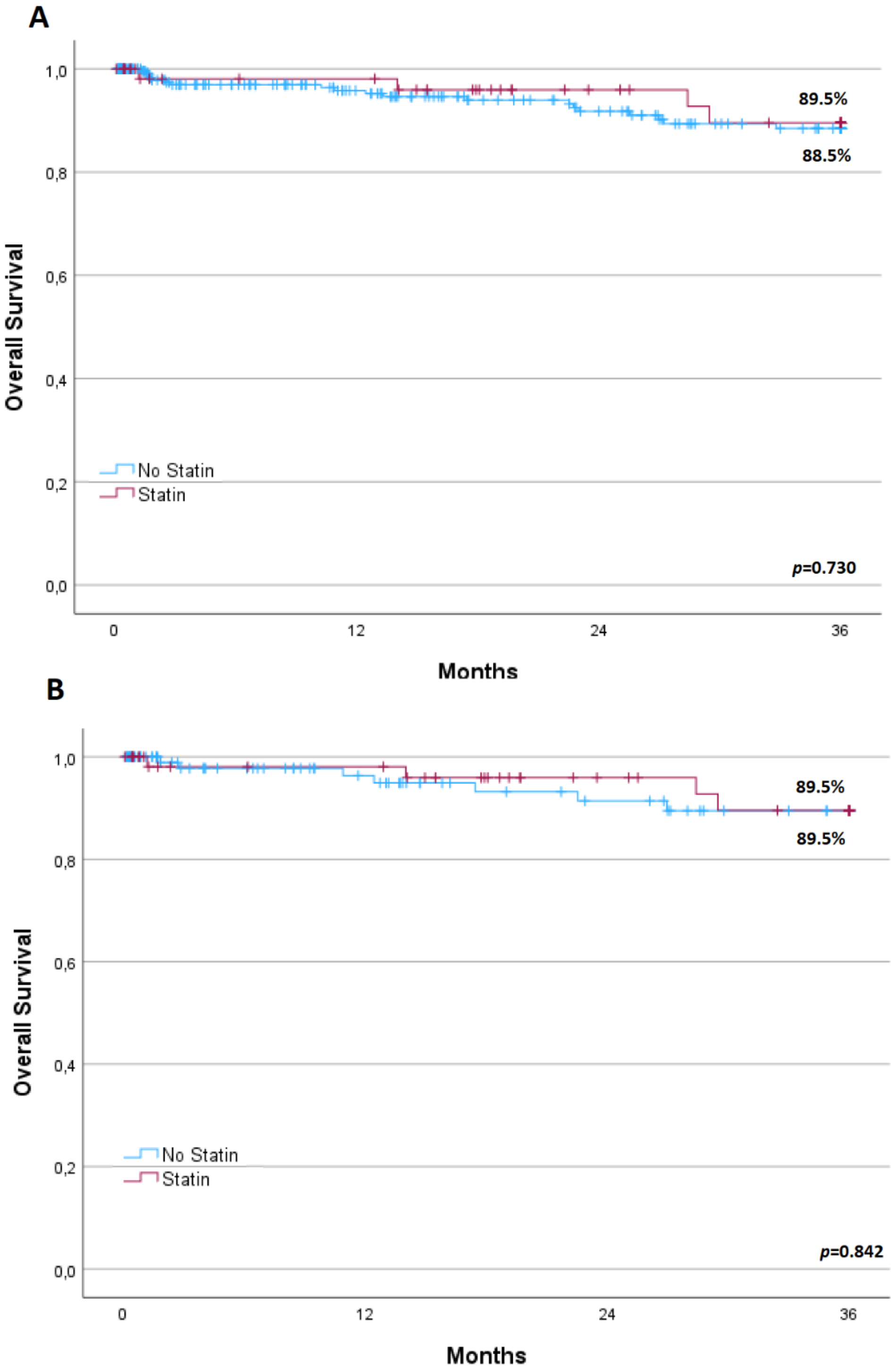

Figure S4. Kaplan–Meier curves comparing recurrence/death free survival between statin users and non-statin users A) in all patients and B) in a matched cohort for statins.

A

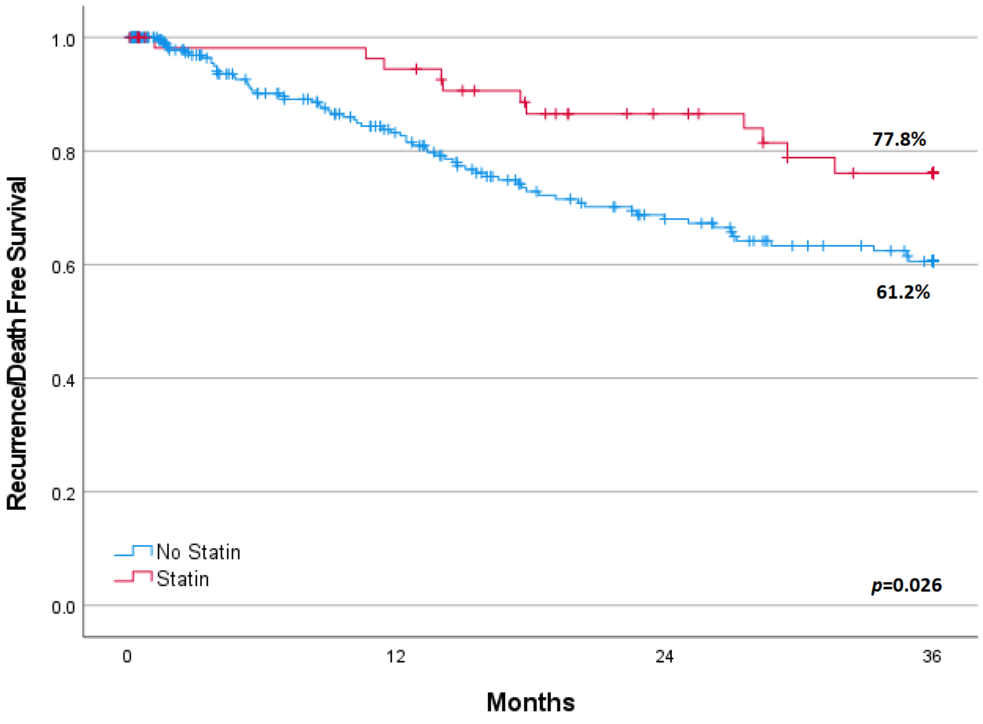

B

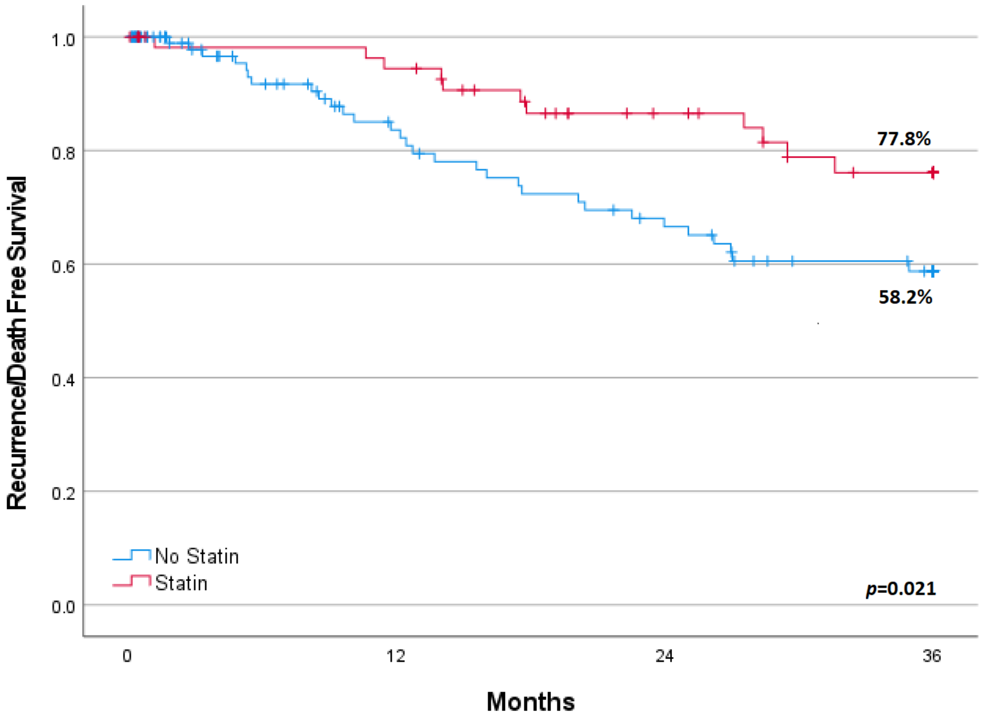

Figure S5. Kaplan–Meier curve comparing recurrence free survival in patients with and without diabetes.

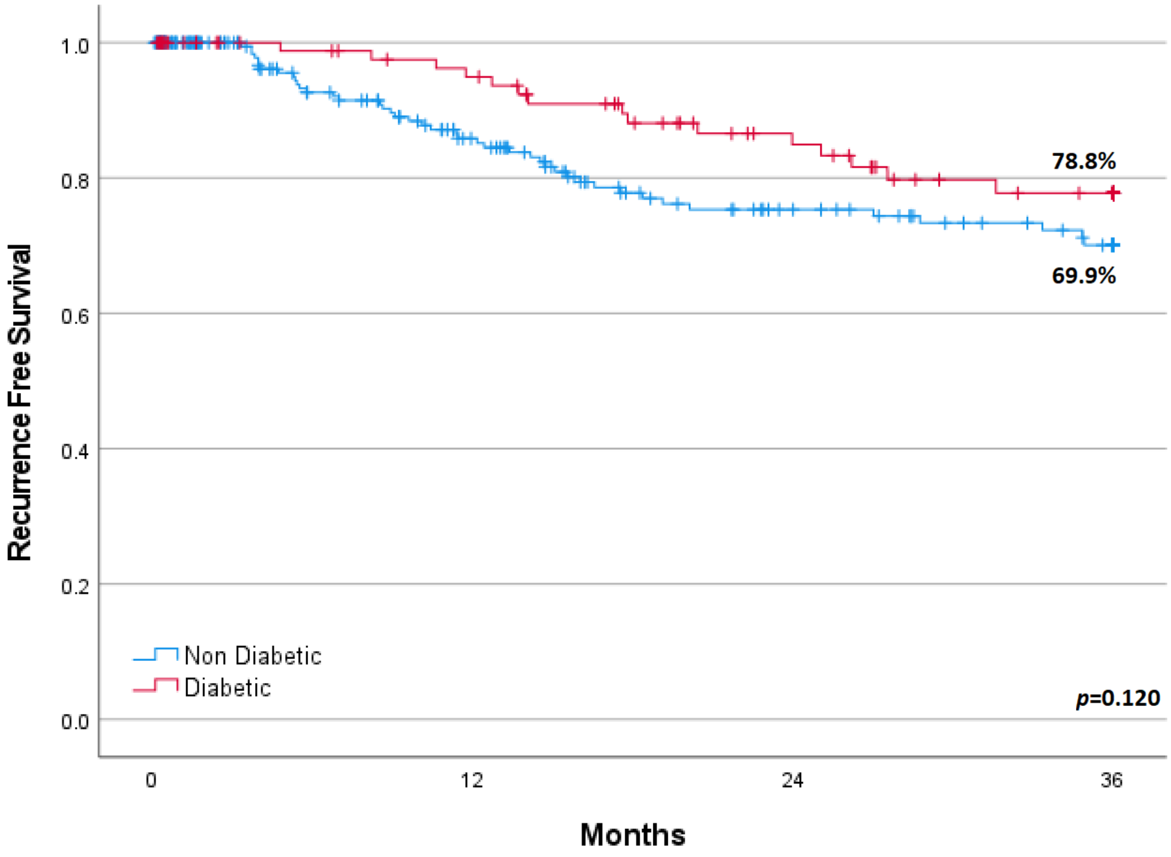

Figure S6. Kaplan–Meier curves comparing recurrence free survival between diabetic metformin users and diabetic non-metformin users A) in all patients with diabetes and B) in a matched cohort in diabetic patients.

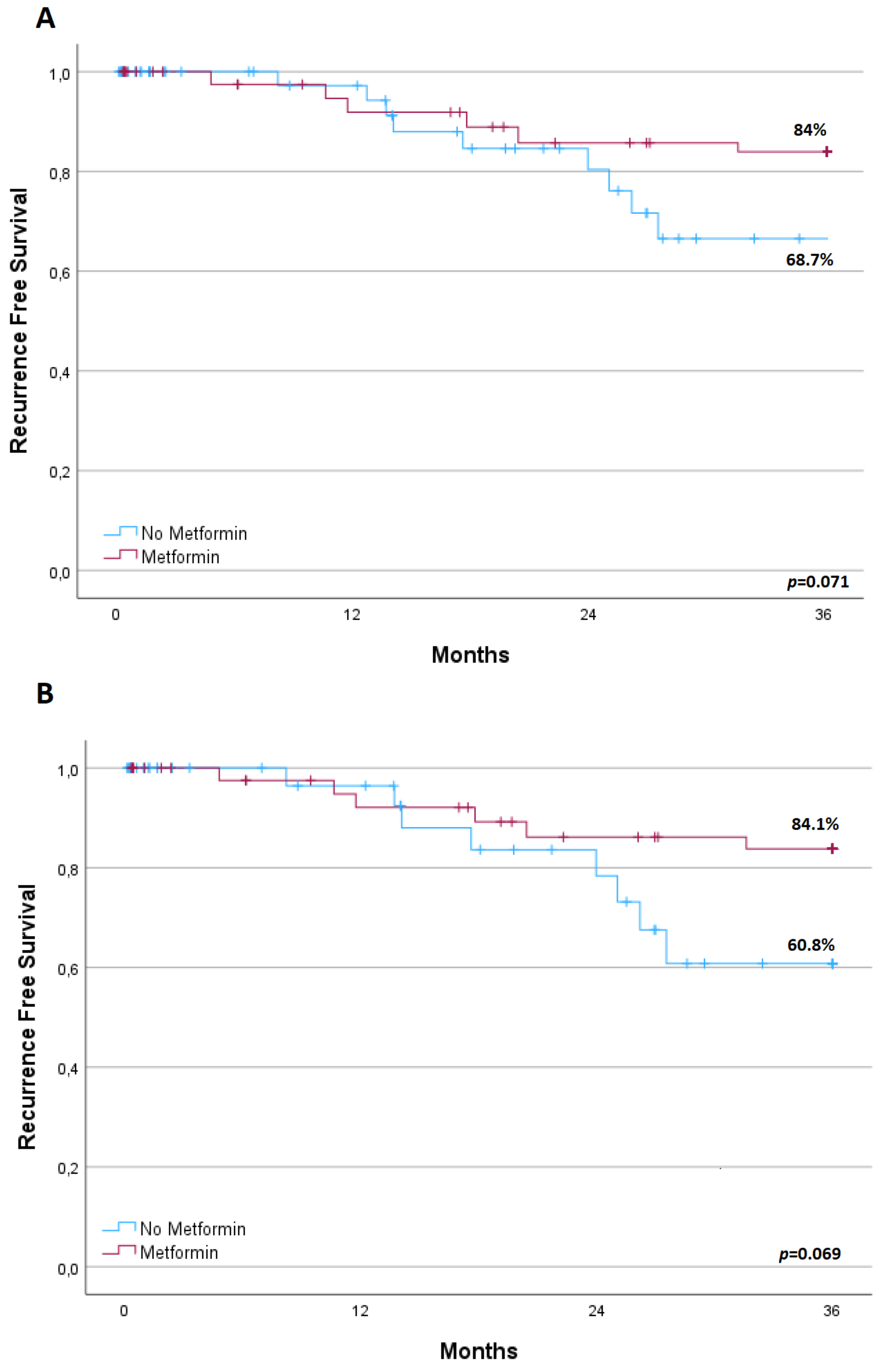

Figure S7. Kaplan–Meier curves comparing cumulative overall survival between metformin users and non-metformin users A) in all patients and B) in a matched cohort for metformin.

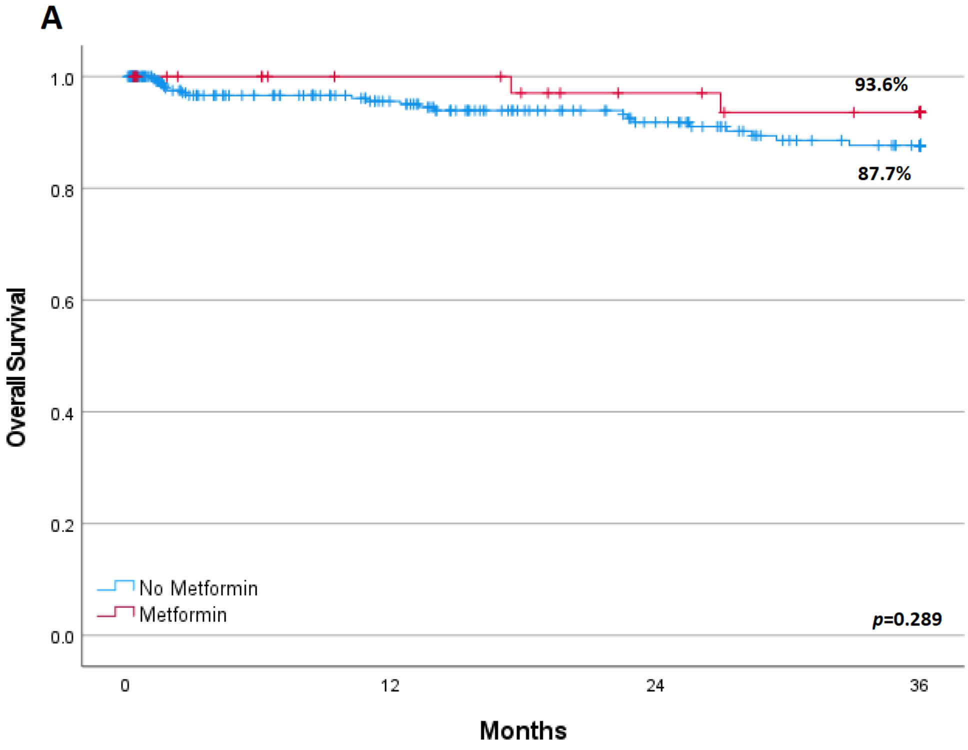

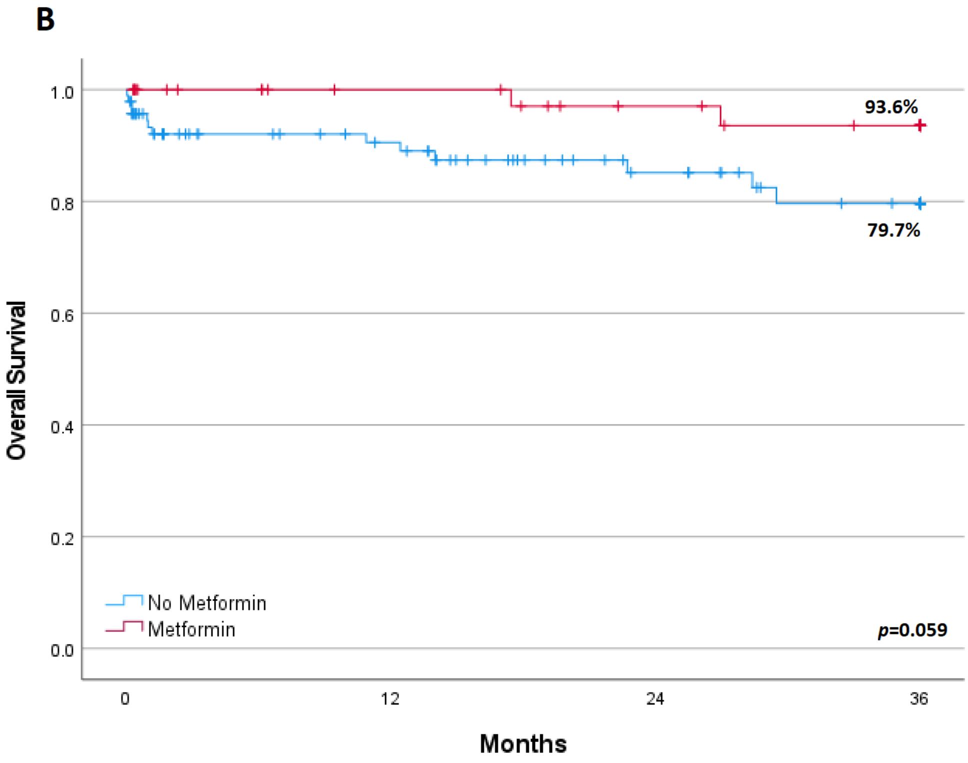

Figure S8. Kaplan–Meier curves comparing recurrence/death free survival between metformin users and non-metformin users A) in all patients and B) in a matched cohort for metformin.

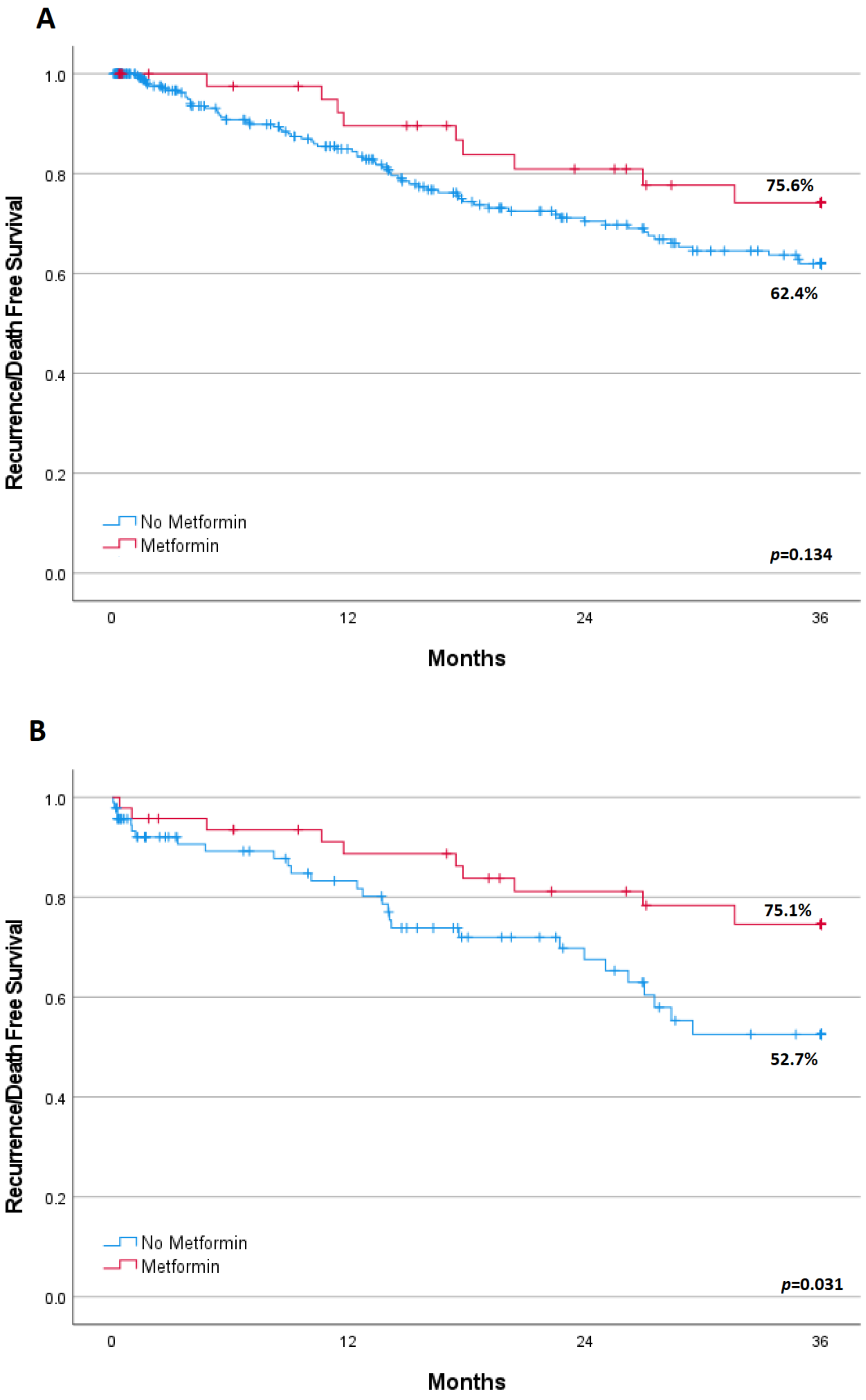

Figure S9. Kaplan–Meier curves comparing recurrence free survival between aspirin users and non-aspirin users A) in all patients and B) in a matched cohort for aspirin.

A

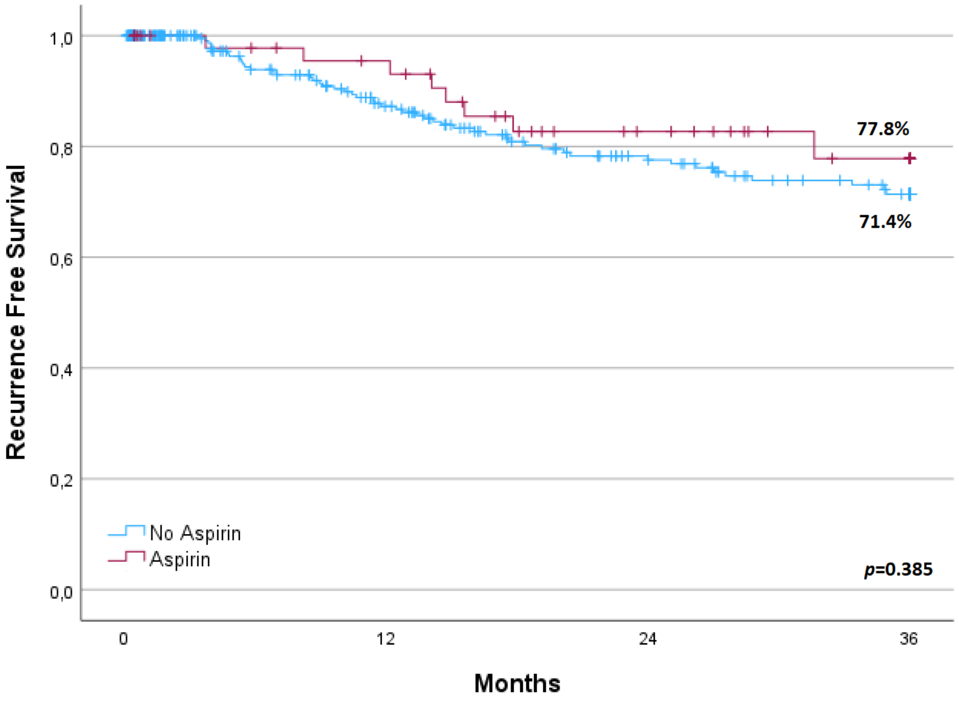

B

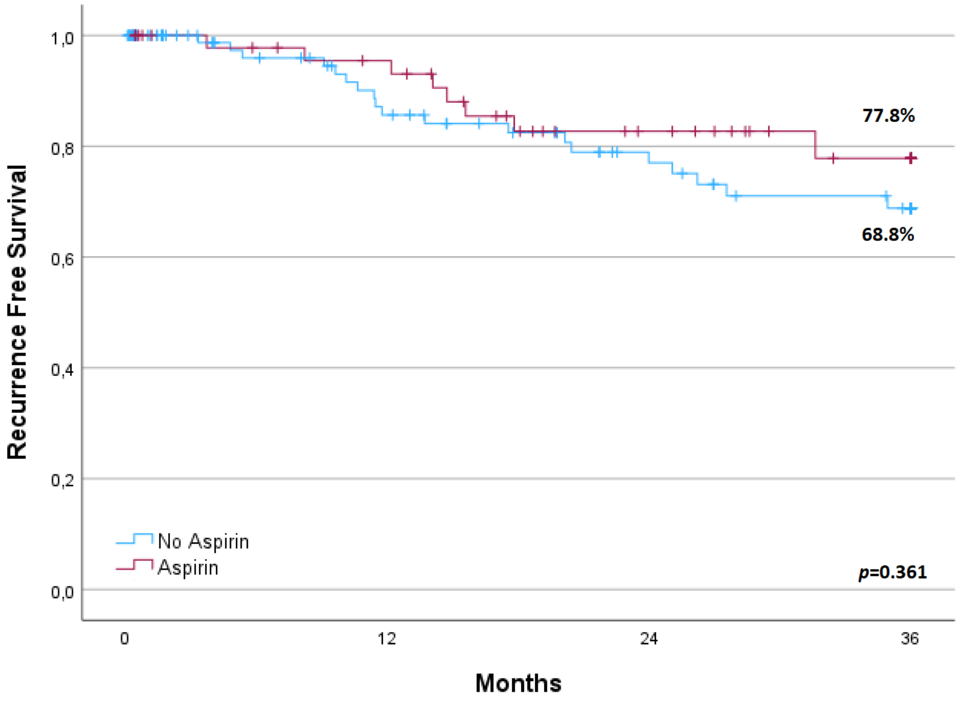

Figure S10. Kaplan–Meier curves comparing cumulative overall survival between aspirin users and non-aspirin users A) in all patients and B) in a matched cohort for aspirin.

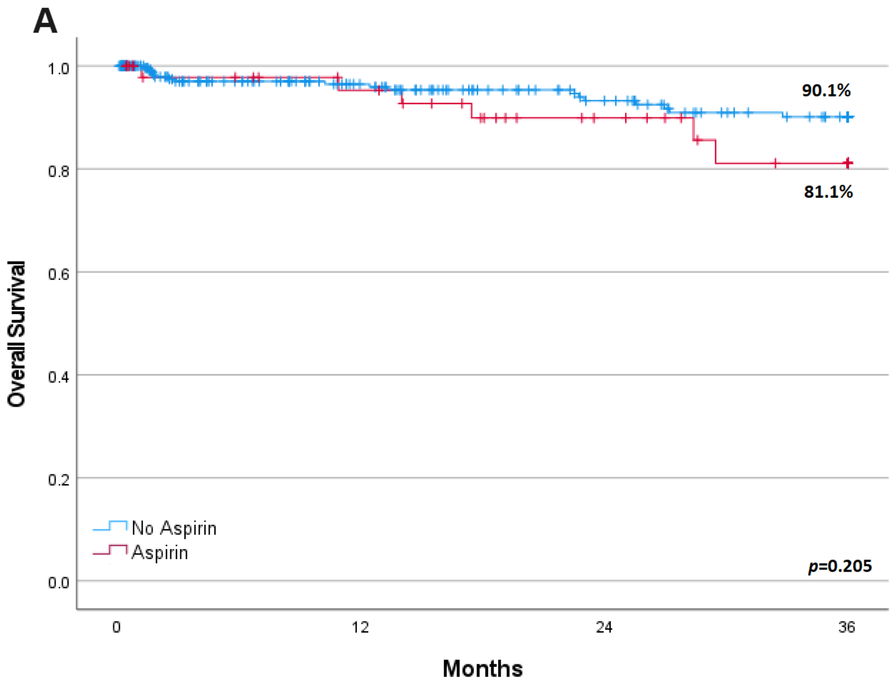

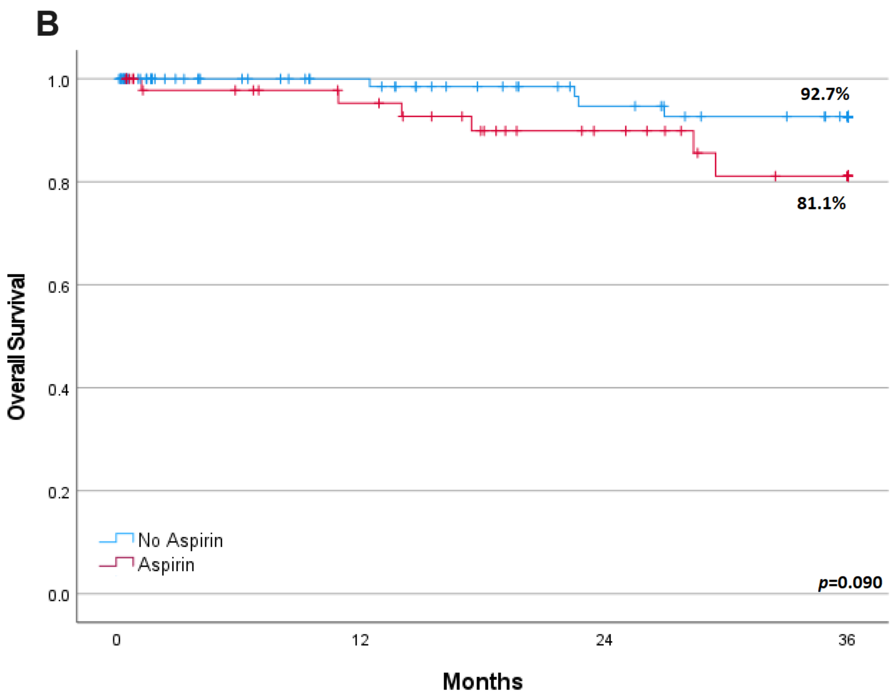

Figure S11. Kaplan–Meier curves comparing recurrence/death free survival between aspirin users and non-aspirin users A) in all patients and B) in a matched cohort for aspirin.

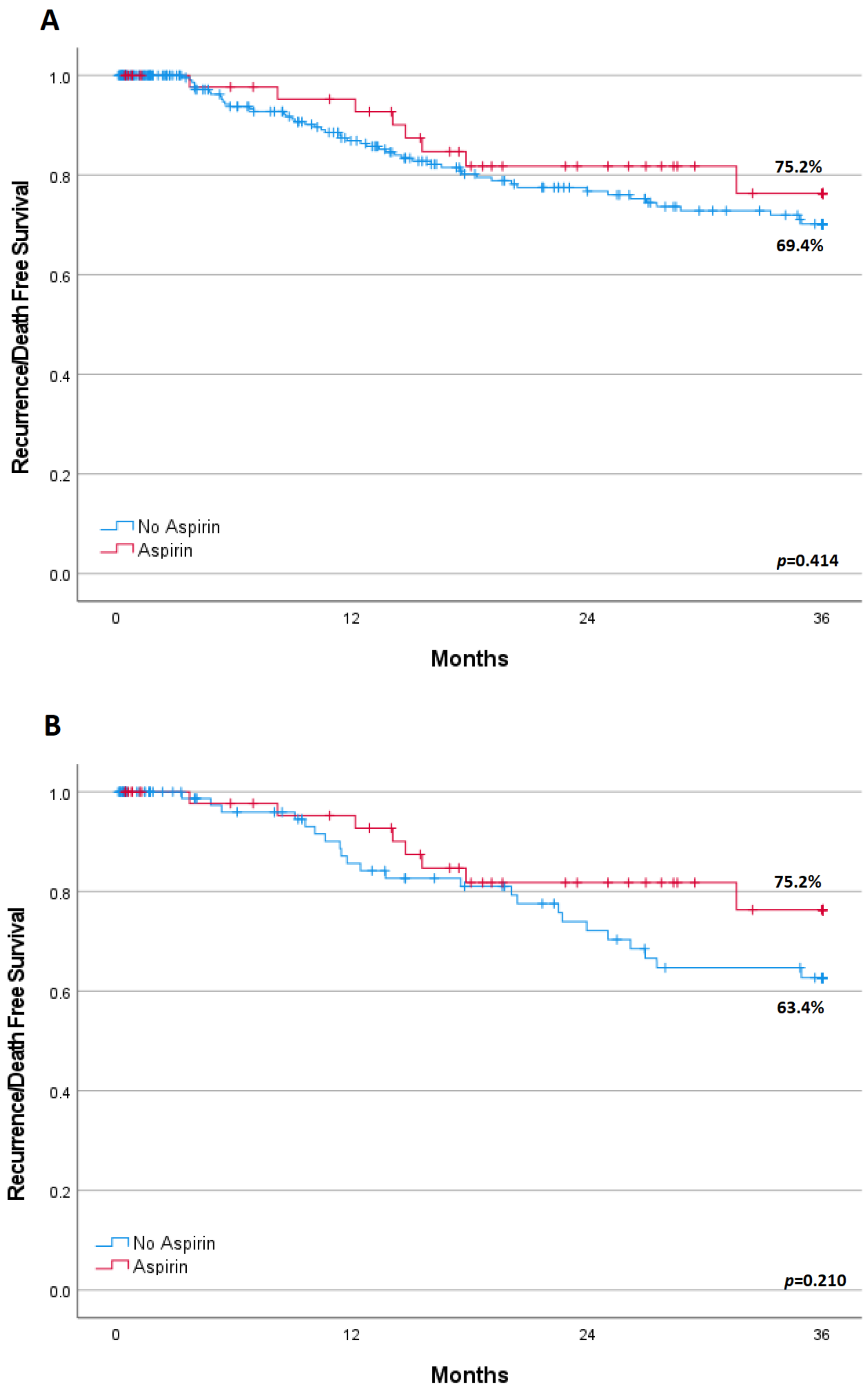

Supplement: Supplementary file 1 — Data S1. Supporting Information. [file CAM4-12-19548-s001.pdf]
